# Uniformly Dispersed and Re-Agglomerated Graphene Oxide-Based Cement Pastes: A Comparison of Rheological Properties, Mechanical Properties and Microstructure

**DOI:** 10.3390/nano8010031

**Published:** 2018-01-09

**Authors:** Wu-Jian Long, Hao-Dao Li, Chang-Le Fang, Feng Xing

**Affiliations:** Guangdong Provincial Key Laboratory of Durability for Marine Civil Engineering, Shenzhen Durability Center for Civil Engineering, College of Civil Engineering, Shenzhen University, Shenzhen 518060, China; 2172332341@email.szu.edu.cn (H.-D.L.); 2150150420@email.szu.edu.cn (C.-L.F.); xingf@szu.edu.cn (F.X.)

**Keywords:** graphene oxide (GO), uniform dispersion, re-agglomeration, rheological properties, hydration products, cement paste

## Abstract

The properties of graphene oxide (GO)-based cement paste can be significantly affected by the state of GO dispersion. In this study, the effects of uniformly dispersed and re-agglomerated GO on the rheological, mechanical properties and microstructure of cement paste were systematically investigated. Two distinct dispersion states can be achieved by altering the mixing sequence: Polycarboxylate-ether (PCE) mixed with GO-cement or cement mixed with GO-PCE. The experimental results showed that the yield stress and plastic viscosity increased with the uniformly dispersed GO when compared to those of re-agglomerated GO cement paste. Moreover, the 3-day compressive and flexural strengths of uniformly dispersed GO paste were 8% and 27%, respectively, higher than those of re-agglomerated GO pastes. The results of X-ray diffraction, Fourier transform infrared spectroscopy and scanning electron microscopy analyses demonstrated that uniformly dispersed GO more effectively promotes the formation of hydration products in hardened cement paste. Furthermore, a porosity analysis using mercury intrusion porosimetry revealed that the homogeneous dispersion of GO can better inhibit the formation of large-size pores and optimize the pore size distribution at 3 and 7 days than the re-agglomerated GO.

## 1. Introduction

Cement-based composites modified by nanomaterial additives have many advantages and have been increasingly used in recent years as engineering materials [1]. These nanomaterials, such as nanofibers, nanosilica and carbon nanotubes, can not only fill the spaces between particles in the C-S-H gel but also act as pozzolans owing to their high surface area-to-volume ratio [2]. However, owing to their small particle size, large specific surface area and high surface energy, the use of such nanomaterials in cement matrices causes high water consumption and agglomeration [3,4]. In previous studies, it was observed that issues with the dispersion of nanomaterials in fresh cement pastes can seriously degrade the properties of the cement [5,6]. Therefore, the problem of dispersing nanomaterials uniformly in a cement matrix is of great importance. The use of graphene oxide (GO) presents many advantages in addressing this problem, as GO is an advanced nanomaterial that exhibits good dispersion in aqueous solutions, promising improvement of the cement pastes in which it is included [7].

GO is derived from graphene that is chemically oxidized and stripped [8], resulting in a single atomic layer that can be expanded at any time in the transverse dimension to tens of microns. The generally accepted structural model of GO is a random distribution of hydroxyl (–OH) and epoxy groups in the GO monolith, with the introduction of carboxyl (–COOH) and carbonyl (C=O) groups along its edges [9]. The functional group has been verified to improve the dispersion of GO in aqueous solution [10,11]. After oxidation, GO becomes an insulated hydrophilic material, resulting in an increase in the interlayer graphene spacing from 0.335 nm to 0.625 nm and an improved dispersion state [12]. 

Based on the predominant performance of cement pastes incorporating GO, it is clear that this additive does not purely serve as a filler. Its modifying effect can be summarized as the nucleation, leading to the aggregation of cement hydration products and the formation of a denser mesh structure, ultimately accelerating hydration and improving early mechanical properties in cement pastes [13]. Therefore, most of the research to date has focused on the effects of the additive content of GO on the cement matrix [14,15,16]. For instance, Lu et al. investigated the small variety of GO reinforced cement-based materials, demonstrating that GO can be used to dramatically enhance the compressive, flexural and tensile strengths of cements [14]. Shang et al. reported that an increase in GO additives reduces the fluidity of cement pastes, while increasing the yield stress and plastic viscosity [15]. Lv et al. have also observed the improvements in the fracture toughness and compressive strength of cement pastes when GO is added [16].

However, the agglomeration of the GO monolithic may be a serious hindrance in the ability of GO to exert its full potential in cement pastes [17]. Although GO is typically well-dispersed in aqueous solutions, there is no guarantee of an equivalently uniform dispersion in a high alkalinity environment due to the steric stabilization of GO with charged ions in a cement pore solution [18]. Because of the complex electrolyte environment of concrete, the dispersion and stability of GO in the pore solution of concrete can be very different from those in aqueous solutions. It is clear that the abundance of calcium cations plays a significant role in the dispersion of GO in fresh cement pastes [19]. Many researchers conducted studies in effort to improve the dispersion of graphene, focusing on the surface modification of graphite during refining, the introduction of foreign molecules such as loading nanoparticles, the addition of surfactant molecules and the use of macromolecules such as aromatic macromolecules, among others [20,21]. Researchers have also investigated the effects of reducing the oxidation of the graphite or the use of the edge of the oxygen-containing functional groups to rely upon electrostatic repulsion to weaken the van der Waals force between GO layers and achieve stable dispersion [22,23]. However, the effects of the state of GO dispersion on the properties of cement-based composites have been rarely studied, although it is critical to the properties of cement pastes whether GO is dispersed uniformly or re-agglomerated during the mixing and casting of cements [24].

Polycarboxylate-ether (PCE) is an attractive superplasticizer for cement-based materials and is generally used as an effective dispersing agent [25]. An appropriate amount of PCE (a PCE/GO wt % of 15) can reduce the agglomeration of GO and enhance the stability of GO in highly alkaline cementitious solutions [26]. The intrinsic mechanism of the synergistic effects between GO and PCE is the carboxylic acid group in PCE that attaches to the surface of GO, decreasing the van der Waals force between GO sheets, dispersing them uniformly throughout the cement matrix [27]. Existing studies showed that the different binding sequence between GO, PCE and cement particles can significantly affect the dispersed state of GO in a cement matrix [28].

To quantify the difference between uniformly dispersed GO and re-agglomerated GO, this study systematically investigates the effects of different dispersed states of GO on the properties of cement pastes, including rheological properties, mechanical properties and microstructure characteristics. To reflect the different dispersion states of GO in cement pastes, two distinct mixing sequences were used to combine GO, PCE and cement. The experimental tests were conducted using advanced characterization techniques, including transmission electron microscopy (TEM), Raman spectroscopy, nuclear magnetic resonance spectroscopy (NMR), X-ray diffraction (XRD), Fourier transform infrared spectroscopy (FT-IR), mercury intrusion porosimetry (MIP) and scanning electron microscopy (SEM).

## 2. Materials and Experimental Procedure

### 2.1. Raw Materials

Type I ordinary Portland cement (OPC), conforming to the requirements of Chinese Standard GB 175 [29], was used as the base material in this research. The chemical composition of the OPC is detailed in Table 1. The graphite oxide used was purchased from the Sixth Element Ltd. (Changzhou, China) and its physical properties are given in Table 2. With the help of probe sonication, a suspension of GO (3 g/L) was prepared by dispersing the graphite oxide powder into water. The frequency and time of ultrasound application was 25 Hz and 2 h, respectively.

Polycarboxylate-ether served as a surfactant to disperse the GO in the cement paste, conforming to the requirements of JG/T 223 [30]. The PCE changes the dispersion of cement grains by increasing the particles distance at the contact point [31,32]. Moreover, the PCE works through dual mechanisms of electrostatic repulsion and steric stabilization, in which the long molecules of the organic polymer wrap around the GO particles, preventing their aggregation by physically blocking them from approaching each other.

### 2.2. Test Methods

#### 2.2.1. Characterization Techniques

The strengths of the hardened cement pastes were tested according to the procedures of GB/T 17671 [33]. The loading speeds used in the compressive and flexural tests were 2.4 kN/s and 50 N/s, respectively.

The characterization of the as-received GO was performed using images of the GO morphology obtained using a JEM-1230 TEM. Scanning electron microscopy (SEM, FEI type, QUANTA FEG 250, FEI Company, Hillsboro, OR, USA) and energy dispersive spectrometry (EDS, EDAX INC, Mahwah, NJ, USA) were used to determine the size of the GO particles, their hybrid morphology and their integration with hydration products.

The phase compositions of the hardened cement paste at different ages with the same dosage of GO were measured by using X-ray diffraction (Bruker D8 ADVANCE type, Bruker Company, Karlsruhe, Germany), which uses Cu tube radiation (k = 1.5418 Å) and 2θ in the range of 2.5–40°. Wide-angle measurements (5–80°) were performed for a duration of 0.2 s per scan.

Raman spectroscopy was conducted at room temperature (20 ± 3 °C) in a Renishaw Via (Renishaw Company, London, UK) apparatus. The spot size was approximately 1 mm, using a 50× objective, 5% of the laser power, 10 s of exposure and three cumulative measurements. The laser wavelength used was 532 nm.

NMR spectroscopy was performed to characterize the specific surface area of the GO using a particle size surface area analyzer (PQ001 type, Niumag Company, Shanghai, China). The samples were vacuumed at 3 mTorr and heated to 80 °C for 5 min prior to analysis. The testing process was divided into two steps: first a solvent (distilled water) measurement was conducted, followed by a sample measurement.

The FT-IR scans were not only used to evaluate the functional groups of the pure GO and cement-based composite but also to monitor the hydration process of the cement paste. The FT-IR spectra of pure GO and the cement-based composite were obtained using a Perkin Elmer Spectrum 100 (Perkin Elmer Company, Waltham, MA, USA) and tested from 600 to 4000 cm^−1^ using 32 scans per measurement.

MIP (Auto Pore IV 9500 type, Germann Instruments Company, Copenhagen, Denmark) was used to examine the size range (0.0060–120 μm) of the cement pore structure. The MIP measurement is based on the principle that the volume of mercury intruding into a porous material depends on the applied pressure. Thus, porosimeters able to detect pressures with pore diameters in the range of 0.006–120 μm were used, corresponding to a maximum and minimum applied pressure of 414 MPa and 345 KPa, respectively.

#### 2.2.2. Characterization of the As-Received Graphene Oxide (GO)

**Figure 1:**
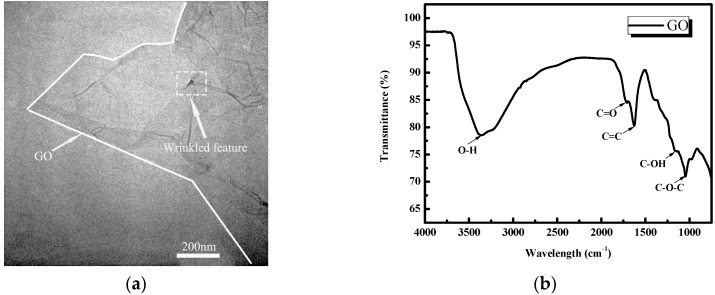

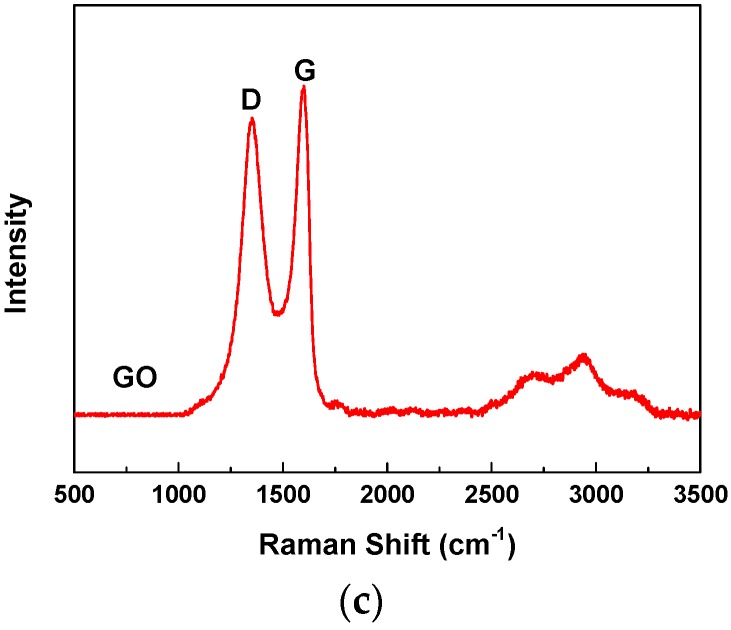
Characterization of graphene oxide (GO) by (**a**) Transmission Electron Microscopy (TEM) Image; (**b**) Fourier transform infrared spectroscopy (FT-IR) transmittance spectra; (**c**) Raman spectra.

Three methods are then used to characterize the GO: TEM, FT-IR spectroscopy and Raman spectroscopy, with the results illustrated in Figure 1a–c, respectively. The TEM image in Figure 1a shows that the GO (right half part of white cure) was an almost transparent nanosheet with various wrinkles and folds. The oxygen-carbon groups in the GO appear in the FT-IR spectrum. Note that the bands appearing at about 3400 cm^−1^ were assigned to the –OH stretching vibration because of the existence of hydrolytic groups and residual water [34]. An absorption peak for the C=C stretch was observed at 1634 cm^−1^ and peaks at 1720 cm^−1^ were also seen in carbonyl groups (C=O), which agrees with the observed peak at 1058 cm^−1^ in the –COOH group, indicating alkoxy (C–O) stretching [35,36]. These oxygen functionalities endow GO with a high hydrophilicity, making it easily dispersible in aqueous solutions. In the Raman spectrum, there are two main Raman shifts characterized by carbon nanomaterials ranging from 1200 to 1700 cm^−1^. The first band at 1620 cm^−1^ can be attributed to the graphite mode (G band), while the second band at 1380 cm^−1^ can be attributed to the diamonded mode (D band) [37]. When compared to graphite, the ID/IG mass ratio is high, with the presence of disordered structure in the graphite arising from the different functional groups in the structure [38,39].

#### 2.2.3. Rheological Measurements

**Figure 2:**
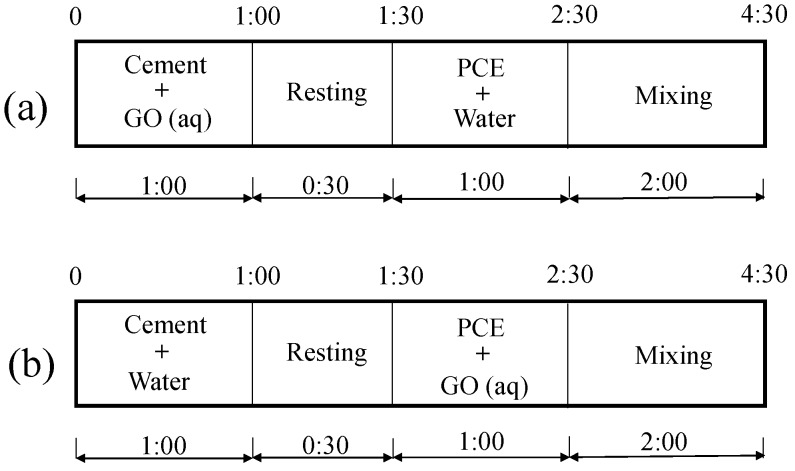
Mixing sequence of suspension or cement paste (unit: min): (**a**) Suspension No. 1 or Paste No. 1 and (**b**) Suspension No. 2 or Paste No. 2.

The rheological parameters of cement paste were measured using an RM 100 touch device manufactured by Lamy Rheology Instruments (Lamy Rheology Instruments Company, Champagne-au-Mont d-’Or, France). A sample tube type DIN 1 and spindle type MK-DIN 2 were used. Before testing, the cement paste was prepared according to Chinese standard GB/T 8077 [40]. The mixing procedure is shown in Figure 2. Immediately after the mixing was concluded, the sample was poured into the sample tube to conduct the rheological measurement. During the rheological test, the shear rate was increased from 3 to 240 s^−1^ over 15 speed intervals. The rheological parameters, apparent viscosity and then shear stress in Pastes No. 1 and No. 2, as defined in Section 2.2.4 below, were measured. The plastic viscosity ($\eta_{p}$) and shear stress ($\tau_{o}$) can be obtained from the slope and intercept of the linear Bingham model relating the shear stress and rate, which can be calculated as follows:

(1) $$\tau =\tau_{o}+\eta_{p}\cdot \gamma ,$$

where τ is the shear stress (in Pa), $\eta_{p}$ is the plastic viscosity (in Pa·s), γ is the shear rate (in s^−1^) and $\tau_{o}$ is the yield stress (in Pa).

If the cement paste exhibits high pseudoplastic or shear thickening behavior, the shear stress might not be calculated, or the obtained value may be lower than the actual value [41]. For this reason, the Bingham model is modified to better extrapolate the shear stress and plastic viscosity under these conditions. The mathematical expression for the Modified Bingham (M-B) model can be elaborated as follows [42,43]:

(2) $$\tau =\tau_{o}+\eta_{p}\cdot \gamma +c\cdot \gamma^{2},$$

where c is a constant.

#### 2.2.4. Mix Proportions

Because the emphasis of the present work is not on the mix proportions of a cement paste but on the dispersion and re-agglomeration behaviors of GO in cement paste before hardening, only the influences of different states of GO on the hydration products of cement were explored. A PCE-to-GO mass ratio of 1:1 was chosen based on preliminary results and is consistent with the limited data found in the literature [44]. The mix proportions of the GO suspension and cement paste are given in Table 3 and Table 4, respectively. In Table 3, the different dispersion states of GO in the suspension are defined as Suspension No. 1 and No. 2. These suspensions are then characterized by NMR. In Table 4, Paste No. 1 and Paste No. 2 are used in the following experimental tests.

The only difference in the mixing sequence of the two samples (shown in Table 3 and Table 4) is whether or not the PCE was pre-mixed with GO before being added into the cement paste. The mixing sequences are shown in Figure 2.

Once the mixing was complete, the paste was poured into a 25-mL tube using a mini-cone to conduct rheological and fluidity measurements. After fluidity measurements, the cement paste was cast into 40 × 40 × 160 mm and 40 × 40 × 40 mm molds for flexural and compressive strength testing, respectively. Custom 10 × 10 × 10 mm molds were used for the MIP and other tests.

## 3. Results and Discussion

### 3.1. Characterization of GO State in the C-GO-PCE Suspensions

**Figure 3:**
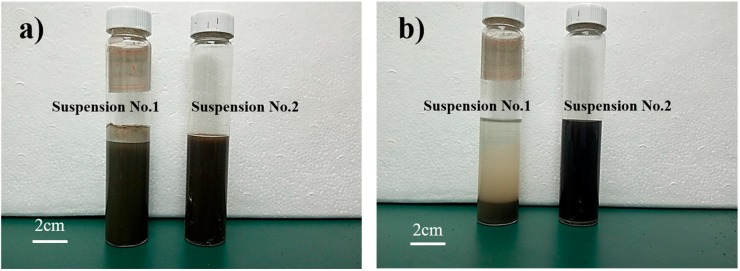
Changes in C-GO-PCE suspensions over time. (**a**) 1 h after mixing; (**b**) 1 day after mixing.

The dispersion state of GO in suspension is characterized using the static precipitation method and the particle size surface characteristic analysis method. Figure 3a,b shows the state of the static suspensions at one hour and one day after mixing, respectively. It can be seen from Figure 3a that most of the GO in Suspension No. 1 has re-agglomerated at one hour after mixing and in Figure 3b that stratification of Suspension No. 1 has clearly occurred, owing to the incorporation of cement particles in the agglomerate. However, with the passage of the time, Suspension No. 2 remains uniformly dispersed and exhibits no re-agglomeration.

To further confirm the dispersion states of the GO in Suspensions No. 1 and No. 2, a 1-mL sample was taken after one day’s static precipitation from the center of the suspension to conduct particle size surface analysis using NMR. The results provided in Table 5 indicate that the specific surface area of GO in Suspension No. 1 was 915.4922 m^2^·g^−1^, while that in Suspension No. 2 was 2217.6372 m^2^·g^−1^. As a result, the majority of GO in Suspension No. 1 would likely re-agglomerate, while the GO in Suspension No. 2 would not. Based on previous studies, the specific surface area in the uniform distribution of GO is 2600 m^2^·g^−1^ [45].

### 3.2. Effect of Different GO Dispersion States on the Rheological Properties of Cement Pastes

**Figure 4:**
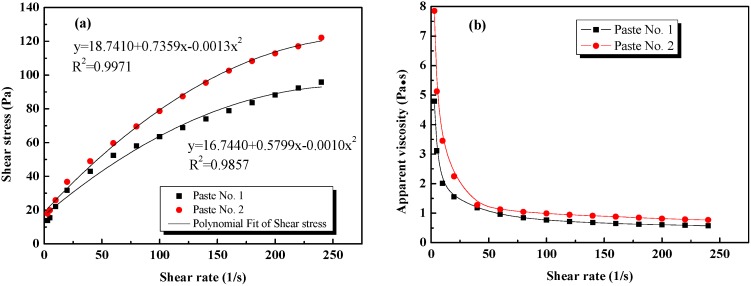
Rheological parameters of GO cement pastes: (**a**) shear rate vs. shear stress; (**b**) shear rate vs. apparent viscosity.

Figure 4 illustrates the variation of corresponding shear stress and apparent viscosity with shear rate. As can be seen in Figure 4a, the fresh cement pastes containing re-agglomerated GO (Paste No. 1) and the uniformly dispersed GO (Paste No. 2) show obvious differences in curve shape but exhibit similar ascending trends prior to solidification, indicating that the two distinct dispersion states of GO in fresh cement pastes evaluated here have a significant effect on the shear stress under different shear rates. Moreover, the shear stress in Paste No. 1 is smaller than that in Paste No. 2 at the same shear rate, indicating that the re-agglomeration of GO may cause the shear stress in the paste to decrease. One of the possible reasons for this is that the surface area of GO decreases after it re-agglomerates, consequently decreasing water absorption such that the free water in the paste increases and then increasing the particle spacing, resulting in an immediate reduction in the friction between particles. Another potential reason for this reduction in shear stress is that the adsorption of PCE on the GO is reduced after it re-agglomerates, causing more of the PCEs to act as a superplasticizer, reducing the amount of water absorbed and promoting an increase in free water in the paste.

It can be seen in Figure 4b that the apparent viscosity of Pastes No. 1 and No. 2 decrease rapidly with the increase in shear rate and eventually it is stabilized. Meanwhile, at the same shear rate, the apparent viscosity of Paste No. 1 is less than that of Paste No. 2 because the flocculation structures in Paste No. 1 are destroyed by the applied shear stress and the re-agglomerated GO is unable to connect any flocculated structures. The degree of flocculation is small in Paste No. 1 and the connection force between structures is extremely weak. In contrast, however, in Paste No. 2, the GO acts to connect flocculated structures, leading to the formation of bigger structures inside the paste, which then leads to greater viscosity and subsequently requires greater shear stress to break up the paste [46].

The yield stress and plastic viscosity of the two cement pastes evaluated in this study are given in Table 6. The results are fitted by the Modified-Bingham (M-B) model, which provides a correlation coefficient close to 0.99, indicating that this fitting model is feasible and appropriate.

### 3.3. Mechanical Properties

Table 7 shows the compressive and flexural strengths of Pastes No. 1 and No. 2. Both the compressive and flexural strengths of Paste No. 1 are lower than those of Paste No. 2. The possible reasons for this are that the uniform dispersion of GO in Paste No. 2 facilitates the hydration process of the cement paste and that the inner pores of the cement matrix are filled with GO nanosheets, increasing the density of the cement matrix and consequently increasing the compressive and flexural strengths of the paste.

It can be seen from Table 7 that the compressive and flexural strengths of Paste No. 2 at 3, 7 and 28 days were increased by 8%, 5% and 4% and 27%, 26% and 19%, respectively, when compared to Paste No. 1. Therefore, it can be concluded that the homogeneous dispersion of GO increases the flexural strength of a paste at an early stage. Similar findings have also been reported by Lu [47]. Clearly, in order to ensure the strength of GO-cement composites, the incorporation of well-dispersed GO into the cement paste must be assured.

### 3.4. Microstructural Properties

#### 3.4.1. Analysis Based on X-ray Diffraction (XRD)

**Figure 5:**
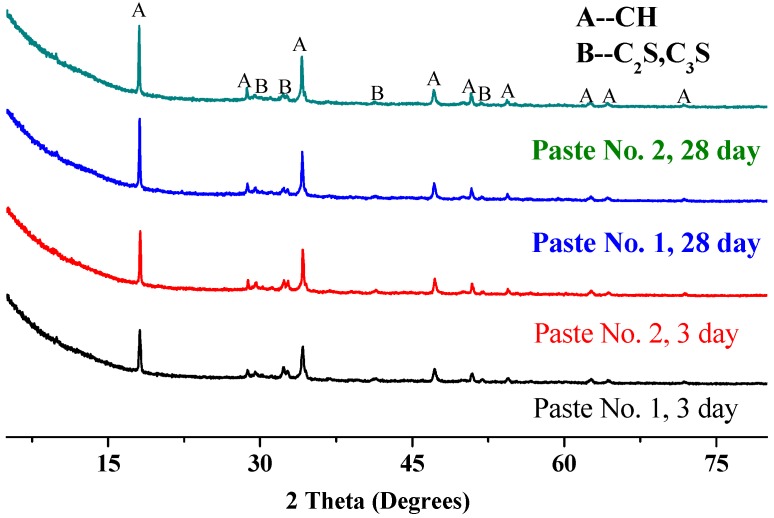
X-ray diffraction (XRD) pattern of pastes (No. 1 and No. 2).

The XRD patterns of Pastes No. 1 and No. 2 at 3 and 28 days are shown in Figure 5. This figure depicts the peaks for the same paste at different ages and for different pastes at the same age as being consistently located, which shows that the formation of calcium hydroxide (Ca(OH)_2_), tricalcium silicate (C_2_S) and dicalcium silicate (C_3_S) crystals is not affected by the state of GO dispersion. However, the magnitudes of these peaks are distinct between pastes and ages. It is seen that the peaks in Paste No. 2 are higher, indicating that the uniform dispersion of GO is more conductive to the quick hydration of cement pastes. The C-S-H gel, generally considered as a dominant source of strength in the hydration product, is amorphous and cannot be detected by XRD analysis but its content can be inferred from the content of calcium hydroxide: a higher content of calcium hydroxide indicates that more C-S-H gels are formed in the hydration product. The peak intensity of calcium hydroxide in Paste No. 2 is stronger than in Paste No. 1, indicating that the content of C-S-H gel in Paste No. 2 is larger. Indeed, the strength of Paste No. 2 is higher than that of Paste No. 1, supporting the likelihood of more C-S-H gels in the paste. Note that the presence of calcium hydroxide, tricalcium silicate and dicalcium silicate in cement paste with added GO was also detected by Shenghua et al. [48]. This indicates that GO can either produce new crystalline structures in hardened cement pastes or transform amorphous C-S-H gel into a crystalline structure. In summary, the presence of re-agglomerated or uniformly dispersed GO does not change the class of hydration products. However, a homogenous dispersion of GO can promote the hydration process of cement pastes.

#### 3.4.2. Analysis Based on FT-IR

**Figure 6:**
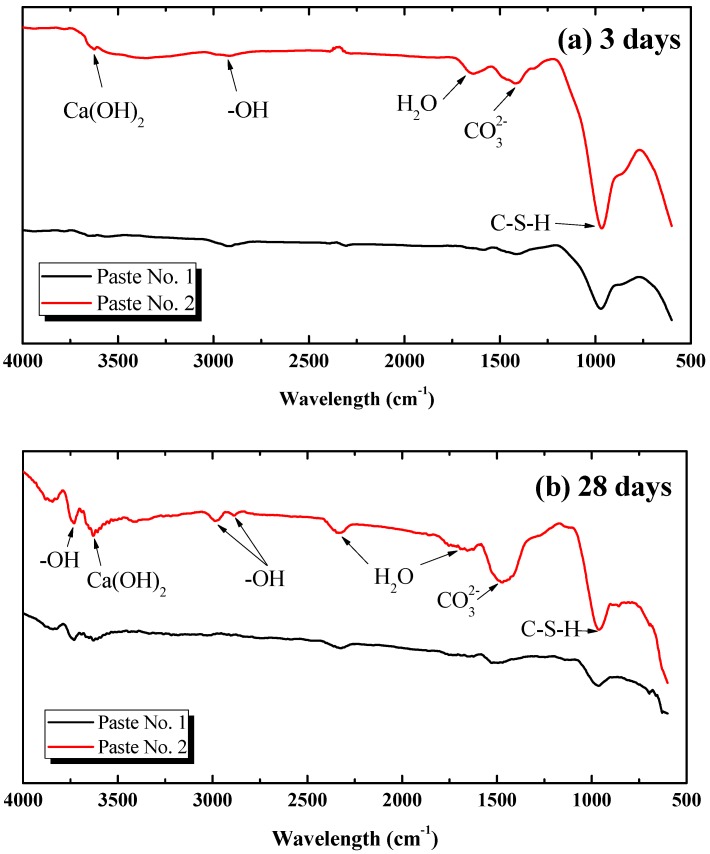
Fourier transform infrared spectroscopy (FT-IR) spectrum of tested pastes at different ages. Variation of wavelength at (**a**) 3 days of curing and (**b**) 28 days of curing.

Figure 6a,b depicts the FT-IR transmission spectra of Pastes No. 1 and No. 2 at 3 and 28 days, respectively, with the spectrum peak reflecting the class of hydration products. In this study, the scanning wavelength is divided into three ranges: the water range (wavelength > 1600 cm^−1^), carbonate range (the wavelength ranges from 1320 cm^−1^ to 1530 cm^−1^) and intrinsic range of the material (wavelength < 1000 cm^−1^). Therefore, wavelengths greater than 1600 cm^−1^ in the spectrum represent the peak absorption of the hydroxyl (–OH bond) existing in the form of different water molecules, with a wavelength around 3620 cm^−1^ representing the absorption peak of calcium hydroxyl. Additionally, a wavelength between 1410 cm^−1^ and 1470 cm^−1^ depicts the absorption peak of the CO_3_^2−^ group, formed by the reaction between Ca(OH)_2_ and CO_2_ in air. The absorption peak at a wavelength of 965 cm^−1^ represents the stretching vibration of the Si–O bond in the C-S-H gel [49].

Combining Figure 5 and Figure 6, the homogenous dispersion of GO (in Paste No. 2) in hardened cement pastes is further illustrated to be capable of promoting the hydration process and generating more C-S-H gels and Ca(OH)_2_ in the cement matrix than a re-agglomerated distribution of GO (in Paste No. 1) at the same curing age. Drawing from Figure 6b, the FT-IR pattern of Paste No. 2 exhibits two extra –OH absorption peaks at the 2990 cm^−1^ and 2890 cm^−1^ wavelengths, suggesting an increase in the presence of water molecules in the cement matrix due to the uniform dispersion of GO.

#### 3.4.3. Analysis Based on Mercury Intrusion Porosimetry (MIP)

**Figure 7:**
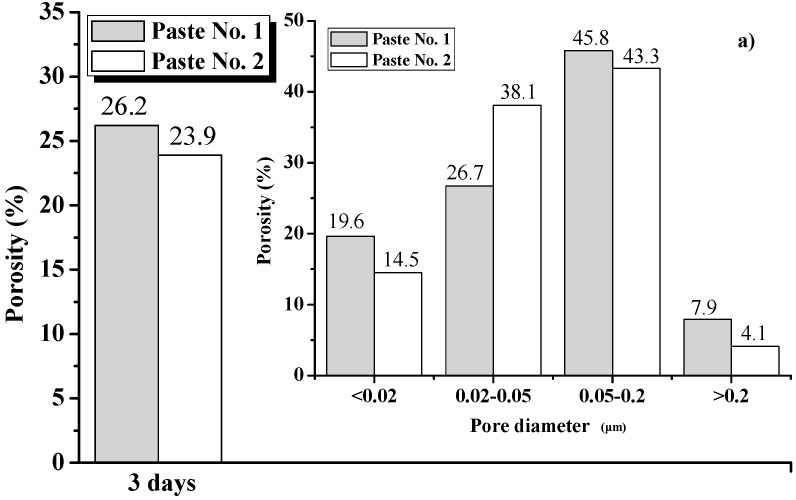

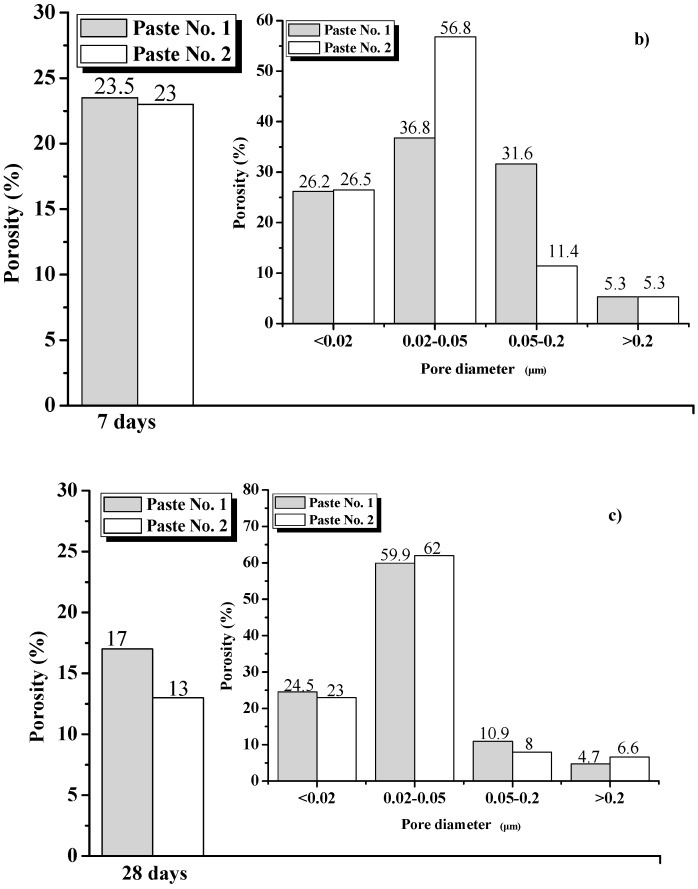
Total porosity of Pastes No. 1 and No. 2 at different ages of: (**a**) 3 days; (**b**) 7 days and (**c**) 28 days, for different pore sizes in the ranges of <0.02 μm, 0.02–0.05 μm, 0.05–0.2 μm and >0.2 μm.

The total porosity and the ratio of different pore sizes to the total porosity for Pastes No. 1 and No. 2 at 3, 7 and 28 days are illustrated in Figure 7. According to the influence of different pore sizes on the properties of concrete, the pores can be divided into innocuous pores (less than 0.02 μm in diameter), less harmful pores (0.02–0.05 μm in diameter), harmful pores (0.05–0.2 μm in diameter) and more harmful pores (greater than 0.2 μm in diameter) [50]. Because both the pore size and pore ratio determine the performance of concrete, increasing the proportion of pores less than 0.05 μm in diameter, rather than solely decreasing the proportion of pores greater than 0.05 μm in diameter, is more favorable for improving the performance of a concrete, particularly in terms of the durability of the materials.

It can be seen from Figure 7 that the total porosity of Pastes No. 1 and No. 2 are 26%, 23.5% and 17% and 23.9%, 23% and 13% at 3, 7 and 28 days, respectively. The total porosity of a sample trends to reduce as the age increases and the total porosities for different ages of Paste No. 1 are consistently greater than those of Paste No. 2, which fits well with the results of strength testing.

Similarly, the percentage of different pore sizes to the total porosity is different for different pastes. This difference reflects the main differentiator in specimen strength. At an age of 3 days, the percentage of pores less than 0.05 μm in diameter in Pastes No. 1 and No. 2 account for 46.3% and 52.6% of the total porosity, respectively, which is consistent with the findings that Paste No. 1 exhibits a lower strength than Paste No. 2.

As the age of the pastes increases from 3 to 7 days, the total porosity of both the Pastes No. 1 and No. 2 decreases, from 26.2% and 23.9% to 23.5% and 23%, respectively. Moreover, the percentage of pores less than 0.05 μm in diameter in Pastes No. 1 and No. 2 increase from 46.3% and 52.6% at 3 days to 63% and 83.3% at 7 days, respectively. This suggests that the increase in strength during this period is due to the pores being continuously filled by hydration products, reducing the total porosity and optimizing the distribution of pore size. Notably, in Paste No. 2 the total porosity remains essentially unchanged, while the ratio of pores less than 0.05 μm in diameter increases from 52.6% to 83.3%. This increase in strength is not a result of a decrease in total porosity but a result of decrease in the percentage of large pore sizes.

Prior to 28 days, the total porosity of Pastes No. 1 and No. 2 decreased from 26.2% and 23.9% to 17% and 13%, respectively and the percentage of pores less than 0.05 μm in diameter increased from 63% and 83.3% to 84.4% and 85%, respectively. Therefore, between the ages of 7 days and 28 days, the strength of Paste No. 1 increased due to the decreases in total porosity and reductions in the percentage of pores less than 0.05 μm in diameter. However, the strength of Paste No. 2 remained higher because the percentage of pores less than 0.05 μm in diameter remains such a significant portion of total porosity.

In summary, hardened cement pastes containing a uniform dispersion of GO have a greater strength than pastes containing re-agglomerated GO. The hydration process of cement pastes containing uniformly dispersed GO is faster than that of cement pastes containing re-agglomerated GO, resulting in a lower cement-based porosity in uniformly distributed pastes at the same age. Additionally, the uniform dispersion of GO is able to increase the ratio of harmless and less-detrimental pores in cement pastes and optimizes the pore size distribution within the matrix, further enhancing the strength of the cement.

#### 3.4.4. Analysis Based on Scanning Electron Microscopy (SEM) and Energy Dispersive Spectrometry (EDS)

**Figure 8:**
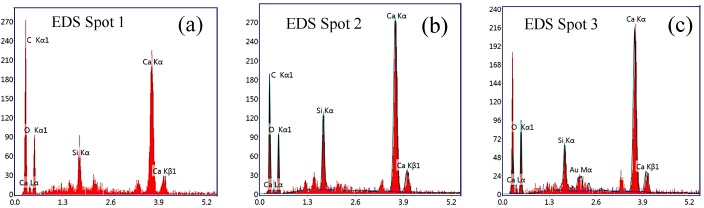

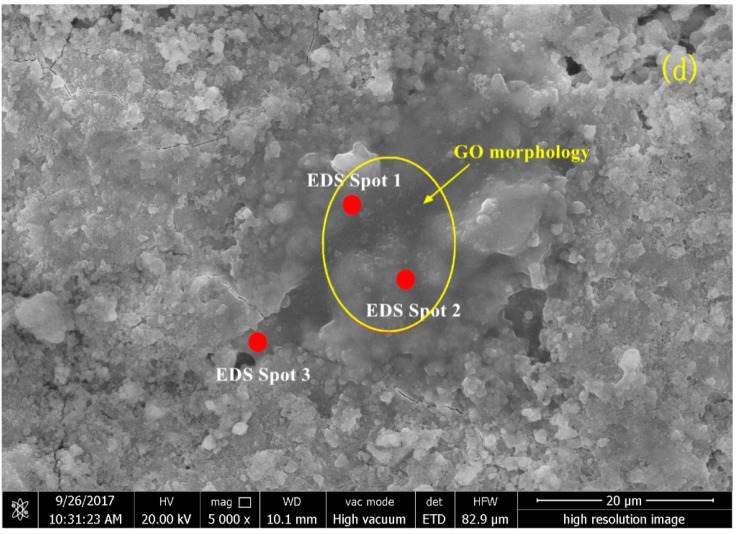
Microstructure analysis of GO-reinforced cement matrix at 28 days: (**a**) and (**b**) EDS for hybrid GO; (**c**) EDS for cement matrix; (**d**) SEM image of dispersion morphology of GO in cement matrix.

Figure 8 shows a SEM image of the dispersion morphology of GO in cement at 28 days, with the combined results from EDS analysis. The image shows that the GOs cover the microcracks in the cement matrix, indicating that the GO not only fills the microcracks but also serves to reinforce the cement matrix. Therefore, the further development of cracks will be constrained. As shown in Figure 8a–c that the elements C, Ca, Si, O and others are found to be present simultaneously, demonstrating that GO integrates well into the cement matrix. Li et al. also reported that chemical bonding occurs between the carboxyl groups in the GO and the C-S-H gel or Ca(OH)_2_ in the cement matrix [51], supporting the finding that GO can be successfully integrated into the cement matrix [52].

### 3.5. Mechanism of GO Reinforcement of Hardened Cement Paste

**Figure 9:**
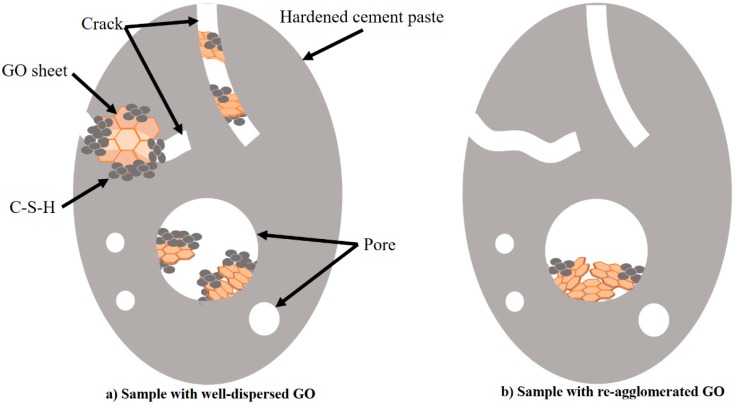
Schematic illustration of GO reinforcement of hardened cement paste: (**a**) sample with well-dispersed GO and (**b**) sample with re-agglomerated GO.

The mechanism through which GO reinforces the cement matrix is illustrated in Figure 9. Based on the abovementioned analysis and discussion, the reinforcing mechanism of uniformly dispersed GO in a hardened cement paste can be attributed to two aspects. First, in view of the layered structure of GO and its compatibility with the cement matrix [53], single and multiple layers of GO can contain or cover microcracks, playing the critical role of restraining crack development while strengthening any weak regions of the cement matrix with the superior tensile strength of GO. Second, GO promotes the hydration process of cement and guides the hydration products such that they form inside the pores of the hardened cement paste. Consequently, the hydration products act to fill the pores, making the substrate denser, as explained by the schematic illustration shown in Figure 9a.

In Figure 9b, the GO nanosheet has re-aggregated and thus only fills the largest pores of the hardened cement paste. Only a small amount of C-S-H gel is produced by hydration inside these pores. Comparing Figure 9a,b, it can be concluded that the mechanical properties of a cement paste are significantly improved by incorporating well-dispersed GO.

## 4. Conclusions

This study has clarified the reinforcing mechanism of GO used in cement paste, which demonstrates that the homogenously dispersed GO can indeed improve the mechanical properties of cement composites. Based on the research findings, the following conclusions are drawn:

(1) The yield stress and plastic viscosity is higher in the cement paste containing well-dispersed GO (Paste No. 2) than in that containing re-agglomerated GO (Paste No. 1). This phenomenon can be attributed to two actions. First, the uniform dispersion of GO increases the water requirement of paste. Second, the GO encourages and enhances the connections between flocculated structures of hydration products, thus makes them larger and more solid.
(2) The addition of GO in cement paste not only can reduce pores in the cement matrix but also can constrain the development of cracks. The comparison of Paste No. 1 and Paste No. 2 demonstrates that the mechanical properties of Paste No. 2, including compressive and flexural strengths, were improved since the uniform dispersion of GO facilitates the hydration process of cement paste and the GO nanosheets fill the inner pores and increase the density of the cement matrix.
(3) The XRD, FT-IR and SEM results show that there was an enhancement in the Ca(OH)_2_ content in Paste No. 2, which is strongly related to the hydration process. Although the hydration products contained in Pastes No. 1 and No. 2 are essentially the same, the hydration process of Paste No. 2 was notably faster. Quantitative analysis by MIP indicates that the total porosity of Paste No. 2 was lower than that of Paste No. 1 and contains lower percentages of harmful pores. Therefore, it can be considered that the uniform dispersion of GO is able to improve the ratio of different pore size and optimize the pore size distribution in cement pastes.
(4) The observed improvement in the mechanical properties of the hardened cement paste accompanying the use of GO is achieved by limiting crack development, reinforcing the weak areas of the cement matrix and promoting C-S-H gel formation inside the pores.

## Figures and Tables

**Table 1:** Chemical compositions of ordinary Portland cement (OPC) (% by mass).

CaO	SiO_2_	Al_2_O_3_	Fe_2_O_3_	SO_3_	MgO	S_r_O	Na_2_O	Cl^−^	Loss on Ignition
62.65	21.88	4.49	3.45	2.44	2.36	0.90	0.51	0.01	1.31

**Table 2:** The properties of graphite oxide.

Appearance	Solid Content (% by Mass)	pH	Viscosity	Absorbance Ratio A230/A600	Carbon (% by Mass)	Molar Ratio (O/C)
Brown paste	43 ± 1	≥1.2	≥2000	≥45	47 ± 5	0.6 ± 1

**Table 3:** Mix proportions of C-GO-PCE suspensions.

Mix No.	Cement (g)	GO (g)	PCE (g)	Water (g)	GO/PCE	pH
Suspension No. 1	2	0.3	0.3	99.7	1:1	12.4
Suspension No. 2	2	0.3	0.3	99.7	1:1	12.4

Notes: (1) The water contained in PCE and GO suspension is incorporated in the calculation of the W/C ratio; (2) C-GO-PCE = cement-GO-PCE.

**Table 4:** Mix proportions of cement pastes.

Mix No.	Cement (g)	Water (g)	W/C	GO (g)	GO/Cement	PCE/GO
Paste No. 1	3600	1440	0.4	2.16	0.06%	1.0
Paste No. 2	3600	1440	0.4	2.16	0.06%	1.0

**Table 5:** Graphene oxide surface parameters and particle size characteristics.

Mix No.	Sample Style (Diameter)	Solvent (Density)	Particle Weight Concentration	Density of Particle (g/mL)	Special Surface (m^2^/g)
Suspension No. 1	GO (350 nm)	Water (1 g/mL)	0.0003	2.2	915.4922
Suspension No. 2	GO (350 nm)	Water (1 g/mL)	0.0003	2.2	2217.6372

**Table 6:** Rheological parameters of cement pastes.

Mix No.	Fitting Equation	$\tau_{o}$ (Pa)	$\eta_{p}$ (Pa·s)	Correlation Coefficient
Paste No. 1	y = 16.7440 + 0.5799x − 0.0010x^2^	16.7440	0.5799	0.9857
Paste No. 2	y = 18.7410 + 0.7359x − 0.0013x^2^	18.7410	0.7359	0.9971

**Table 7:** Compressive and flexural strengths of cement pastes (in MPa).

Age (Days)	Compressive Strength (% Increase)		Flexural Strength (% Increase)	
	Paste No. 1	Paste No. 2	Paste No. 1	Paste No. 2
3	43.1	46.7 (8%)	2.2	2.8 (27%)
7	58.5	62.0 (5%)	5.0	6.3 (26%)
28	62.3	64.8 (4%)	7.5	8.9 (19%)
